# Markers of B-lymphocyte activation are elevated in patients with early rheumatoid arthritis and correlated with disease activity in the ESPOIR cohort

**DOI:** 10.1186/ar2773

**Published:** 2009-07-23

**Authors:** Jacques-Eric Gottenberg, Corinne Miceli-Richard, Béatrice Ducot, Philippe Goupille, Bernard Combe, Xavier Mariette

**Affiliations:** 1 Rhumatologie, Hôpitaux Universitaires de Strasbourg, Centre de Référence National des Maladies Auto-Immunes Systémique Rares, EA 3432 « Physiopathologie des Arthrites », Strasbourg, France; 2 Institut Pour la Santé et la Recherche Médicale (INSERM) Unité 822, 'Epidémiologie, Démographie et Sciences Sociales', IFR69; Institut National d'Etudes Démographiques (INED); Université Paris 11, Le Kremlin-Bicêtre, France; 3 CHRU de Tours; UMR CNRS 6239, Université de Tours; INSERM CIC-202, Tours, France; 4 Immuno-Rhumatologie, Hôpital Lapeyronie, Université Montpellier 1, UGM 5535, Montpellier, France; 5 Rhumatologie, INSERM U802, Université Paris-Sud 11, Hôpital Bicêtre, Assistance Publique-Hôpitaux de Paris (AP-HP), Le Kremlin Bicêtre, France

## Abstract

**Introduction:**

Little is known about systemic B-cell activation in early rheumatoid arthritis (RA). We therefore evaluated the serum levels of markers of B-cell activation in patients included in the ESPOIR early arthritis cohort.

**Methods:**

In the ESPOIR early arthritis cohort (at least 2 swollen joints for more than 6 weeks but less than 6 months), 710 patients were assessed at 1 year and either met the 1987 American College of Rheumatology criteria for RA (n = 578) or had undifferentiated arthritis (n = 132). Baseline serum samples of patients naïve to corticosteroid and disease-modifying antirheumatic drug treatment were assessed for beta2-microglobulin, IgG, IgA, IgM, immunoglobulin free light chains of immunoglobulins, and B-cell activating factor of the tumor necrosis factor family (BAFF). The BAFF gene 871T>C polymorphism was genotyped in all patients.

**Results:**

All markers of B-cell activation except BAFF and IgM were significantly higher in patients with early RA than those with undifferentiated arthritis. Anti-cyclic citrullinated peptide (anti-CCP) and beta2-microglobulin were associated with a diagnosis of early RA in the multivariate analysis. Markers of B-cell activation, except BAFF, were associated with disease activity, rheumatoid factor and anti-CCP secretion. The BAFF gene polymorphism was not associated with early RA.

**Conclusions:**

Markers of B-cell activation are elevated in patients with early RA, compared with undifferentiated arthritis, independently of any systemic increase in BAFF secretion, and correlate with disease activity. This study sheds new light on the early pathogenic role of B-lymphocytes in RA and suggests that targeting them might be a useful therapeutic strategy in early RA.

## Introduction

For decades – ever since the discovery of rheumatoid factor (RF) – B cells have been known to play a pathogenic role in established rheumatoid arthritis (RA) [1-4]. More recently, the secretion of RF and antibodies against cyclic citrullinated peptide (anti-CCP) was demonstrated to precede RA clinical onset by many years [5,6], which suggests that activation of autoreactive B cells might be an early pathogenic event. However, very little is known about the activation of alloreactive B cells in patients with early RA. Markers of B-cell activation, such as beta2-microglobulin, immunoglobulin levels, free light chains (FLCs) of immunoglobulins, and BAFF (B-cell activating factor of the tumor necrosis factor [TNF] family) – which are all elevated in established RA [7-10] – could be useful in determining the extent of B-cell activation in early RA. One of the objectives of the French multicenter prospective cohort, ESPOIR [11], is to determine the specific biological features of early RA by comparing serum samples from patients with either early RA or other early arthritis who are naïve to disease-modifying antirheumatic drugs (DMARDs) and steroids. In this study, we assessed baseline levels of several markers of nonspecific B-cell activation, such as beta2-microglobulin, immunoglobulin FLCs, IgG, IgA, and IgM as well as serum BAFF. Since the BAFF 871T>C polymorphism is reported to be correlated with serum BAFF level in various diseases [12-14], patients were also genotyped for this polymorphism. Our findings show that baseline serum markers of B-cell activation are higher in patients with early RA than in patients with undifferentiated arthritis (UA). In addition, their increase is correlated with disease activity but is independent of serum BAFF levels and the BAFF gene polymorphism.

## Materials and methods

### Patients

The French multicenter prospective cohort of patients with early arthritis (ESPOIR) has included 813 patients with early arthritis between December 2002 and March 2005 and plans to follow them for 10 years. Patients were eligible for inclusion in the cohort if they had a definitive or probable clinical diagnosis of RA or a diagnosis of UA with a potential for progressing to RA. Thus, these patients had at least two swollen joints, present for more than 6 weeks but less than 6 months, and were naïve for DMARDs and corticosteroids at inclusion. Their baseline clinical, immunological, and radiological features were recently published [11,15]. Eighty-three patients missed the 1-year visit and were not included in the present study. Twenty patients fulfilling American College of Rheumatology (ACR) or international consensus group criteria for other arthritides were excluded. Diagnosis of RA was defined after 1 year of follow-up, according to cumulative 1987 ACR criteria for RA (independently from the positivity for anti-CCP). Patients without any definite diagnosis until the 1-year follow-up visit were diagnosed with UA. The present study thus analyzes the 710 patients who completed the first three visits (at baseline, 6 months, and 1 year) and were diagnosed as having RA or UA after 1 year of follow-up. Serum samples from 80 healthy blood donors were assessed for BAFF levels, and DNA samples from 90 healthy blood donors were assessed for the BAFF gene polymorphism. The Montpellier Ethics Committee approved the study in July 2002, and all patients and controls provided informed consent.

### Serum assessments

Serum samples were collected at enrollment and immediately stored at -80°C. One biological resource center was in charge of centralizing and managing biological data collection. Assessments of serum beta2-microglobulin, IgG, IgA, IgM, immunoglobulin FLCs (nephelometry), and BAFF (enzyme-linked immunosorbent assay, or ELISA; R&D Systems, Lille, France) were centralized. Serum samples of 40 patients were simultaneously thawed daily, and all of their markers of B-cell activation were assessed that day. Serum measurements of IgM and IgA RF (ELISA; Menarini France, Rungis Cedex, France, positive >9 IU/mL) and anti-CCP (anti-CCP2; DiaSorin, Saluggia [Vercelli], Italy, positive >50 U/mL) levels were performed in a central location and determined as previously reported [11].

### DNA genotyping

After genomic DNA was isolated from peripheral blood mononuclear cells, the BAFF promoter gene polymorphism 871T>C was genotyped with competitive allele-specific polymerase chain reaction by using FRET (fluoroscence resonance energy transfer) technology. This genotyping was successful for 686 patients and 90 healthy blood donors.

### Radiological data

At enrollment, radiographs of the hands and wrists (anteroposterior view) and of the feet (anteroposterior and oblique views) were taken. Their interpretation was standardized as described previously [11,15].

### Statistical analysis

Continuous data are presented as medians with interquartile ranges. In spite of the high number of patients and because of the uneven frequency of early RA and UA data, we used nonparametric tests for statistical analysis of continuous variables. The Mann-Whitney *U *test was used to compare continuous data between patients with RA and patients with UA or, within the RA group, between patients with or without RF, anti-CCP, or radiological erosions. The chi-square test was used to compare BAFF allele and genotype frequencies between patients with RA, patients with UA, and controls. The chi-square test was also used to compare the proportion of anti-CCP between RA patients and UA patients and, within the RA group, to compare the proportion of RF and anti-CCP between patients with and without early erosions. Correlations between disease activity score using 28 joint counts (DAS28) or Health Assessment Questionnaire (HAQ) and markers of B-cell activation were studied with the Spearman rank test. Biological markers associated on univariate analysis with type of arthritis (RA or UA: erythrocyte sedimentation rate [ESR], C-reactive protein [CRP], anti-CCP, IgG, IgA, beta2-microglobulin, and kappa and lambda FLCs of immunoglobulins) or, in RA, with the presence of initial erosions (IgM-RF, IgA-RF, anti-CCP, ESR, IgA, and kappa FLCs of immunoglobulins) were included in multivariate logistic regression models. Statistical analyses were performed with Stata SE 9.2 (StataCorp LP, College Station, TX, USA).

## Results

### Characteristics of the study population

The median age of the 710 patients (76.5% of whom were female) was 49 (40 to 64) years. At enrollment, 48.3% of the patients were IgM-RF-positive and 40.0% were anti-CCP-positive. Median DAS28 was 5.1 (4.3 to 5.9), and median HAQ was 0.9 (0.4 to 1.4). Radiographic erosions on hands or feet were seen at inclusion in 22.5% of the patients. Five hundred seventy-eight patients (79.2%) had met the 1987 ACR criteria for RA at any time since their inclusion and were classified as having early RA, and 132 patients still had UA at the 1-year follow-up visit. Baseline characteristics of the 710 patients are summarized in Table 1.

**Table 1:** Baseline characteristics of 710 patients with early rheumatoid arthritis or undifferentiated arthritis

	Patients with early RA or UA	Early RA	UA	Univariate analysis: *P *value	Multivariate analysis: OR (95% CI), *P *value
		(1)	(2)	(1) versus (2)	(1) versus (2)
	n = 710	n = 578	n = 132		
Percentage of females	76.5%	77%	74%	0.8	
Age, years	49.0 (40.0–64.0)	50.1 (42.3–65.2)	47.4 (39.5–62.4)	0.6	
ESR, mm at 1st hour	22.0 (11.2–38)	23.0 (12.0–40.1)	17.0 (10.0–32.0)	0.01	0.9 (0.9–1.0), 0.6
CRP, mg/L	9.0 (0–24.0)	9.5 (3.0–25.0)	6.0 (0–16.0)	0.002	1.0(0.9–1.1), 0.3
Positivity for anti-CCP	40.3%	47.4%	13.2%	<0.0001	5.8 (3.4–10.0), 0.001
BAFF, ng/mL	0.7 (0.5–1.0)	0.7 (0.5–1.0)	0.7 (0.5–0.9)	0.5	
Beta2–microglobulin, mg/L	1.9 (1.7–2.3)	2.0 (1.7–2.4)	1.8 (1.6–2.1)	0.0002	1.5 (1.0–2.0), 0.03
Total IgG, g/L	13.5 (11.5–16.1)	13.5 (11.7–16.1)	12.9 (10.9–16.0)	0.03	0.9 (0.9–1.0), 0.4
Total IgA, g/L	2.6 (1.9–3.5)	2.8 (1.9–3.5)	2.2 (1.7–3.1)	<0.0001	1.2 (0.9–1.5), 0.06
Total IgM, g/L	1.5 (1.1–2.0)	1.5 (1.1–2.0)	1.4 (1.1–1.9)	0.5	
Kappa free light chain, mg/L	13.8 (10.5–17.9)	14.3 (10.9–18.7)	11.7 (9.6–14.9)	<0.0001	1.0 (0.9–1.0), 0.6
Lambda free light chain, mg/L	16.4 (12.8–21.6)	17.5 (13.2–22.5)	14.5 (11.6–18.8)	<0.0001	0.9 (0.9–1.0), 0.9
Ratio of kappa to lambda	0.8 (0.3–0.99)	0.8 (0.7–1.0)	0.8 (0.7–0.9)	0.9	

Results are expressed as median values (25th–75th values) or as percentages. Comparison of proportions of presence of anti-cyclic citrullinated peptide (anti-CCP) used chi-square test, and comparison of the other variables used Mann-Whitney *U *test. Baseline markers associated on univariate analysis with rheumatoid arthritis (RA) diagnosis were analyzed in multivariate analysis. BAFF, B-cell activating factor of the tumor necrosis factor family; CI, confidence interval; CRP, C-reactive protein; ESR, erythrocyte sedimentation rate; OR, odds ratio; UA, undifferentiated arthritis.

### Elevated markers of B-cell activation in patients with early rheumatoid arthritis

Comparison of markers of B-cell activation, except RF, which belongs to ACR criteria used to define RA, was performed between patients with early RA and patients with UA. Patients with early RA had significantly elevated serum levels of beta2-microglobulin, IgG, IgA, and immunoglobulin kappa and lambda FLCs. Anti-CCP, ESR, and CRP were also associated on univariate analysis with the diagnosis of early RA (Table 1). In the multivariate analysis, which included all markers associated with RA on univariate analysis, diagnosis of RA after 1 year of follow-up was associated with levels of anti-CCP (odds ratio [OR] 5.8 [3.4 to 10.0], *P *= 0.001) and beta2-microglobulin (OR 1.5 [1.0 to 2.0], *P *= 0.03) and tended, though not significantly, to be associated with IgA levels (OR 1.2 [0.9 to 1.5], *P *= 0.06).

### Serum BAFF levels in patients with early rheumatoid arthritis and with undifferentiated arthritis

BAFF was a possible explanation for the higher levels of markers of B-cell activation in early RA. The median BAFF levels were 0.7 (0.5 to 1.0) ng/mL in the 710 patients with arthritis and 0.5 (0.4 to 0.6) ng/mL in the 80 healthy blood donors (*P *<0.0001). However, BAFF levels were similar in patients with early RA (0.7 [0.5 to 1.0]) and those with UA (0.7 [0.5 to 0.9], *P *= 0.5) (Table 1).

### Association of the BAFF 871T>C gene promoter polymorphism with early rheumatoid arthritis and undifferentiated arthritis

We analyzed the distribution of the BAFF 871T>C polymorphism among patients with early RA and patients with UA. The two groups did not differ significantly for any allelic or genotypic frequencies (Table 2). Likewise, the allelic and genotypic frequencies in early RA patients did not differ significantly from those in healthy controls. This polymorphism was not associated with any baseline characteristic of early RA: DAS28 (*P *= 0.46), erosive arthritis (*P *= 0.2), RF (*P *= 0.9), and anti-CCP (*P *= 0.2). BAFF levels were similar in the early RA patients regardless of genotype for the BAFF 871T>C polymorphism (*P *= 0.2). No BAFF gene polymorphism was associated with serum BAFF level in the overall patient cohort either (*P *= 0.1).

**Table 2:** Allelic and genotypic distribution of BAFF -871T/C among patients with early rheumatoid arthritis, undifferentiated arthritis, and controls

BAFF -871T/Caia	Early RA	UA	Controls	*P *value	Odds ratio (95% CI)	*P *value	Odds ratio (95% CI)
Allele frequencies	n = 1,112	n = 260	n = 180		Early RA versus UA		Early RA versus controls
-871C (%)	603 (54)	146 (56)	90 (50)	NS	1.00 (0.77–1.30)	NS	1.18 (0.86–1.62)
-871T (%)	509 (46)	114 (44)	90 (50)				
							
Genotype frequencies	n = 556	n = 130	n = 90				

CC (%)	171 (31)	43 (33)	21 (23)	NS	1.02 (0.68–1.53) CC versus CT and TT	NS	1.46 (0.87–2.46) CC versus CT and TT
CT (%)	261 (47)	60 (46)	48 (54)				
TT (%)	124 (22)	27 (21)	21 (23)	NS	1.02 (0.66–1.60) TT versus CT and CC	NS	0.94 (0.56–1.60) TT versus CT and CC

Comparisons used chi-square test. BAFF, B-cell activating factor of the tumor necrosis factor family; CI, confidence interval; NS, not significant; RA, rheumatoid arthritis; UA, undifferentiated arthritis.

### Association of markers of B-cell activation with initial disease activity, Health Assessment Questionnaire, autoantibody secretion, and early erosion in patients with early rheumatoid arthritis

We subsequently investigated whether an elevated level of markers of B-cell activation was associated with a specific clinical, immunological, or radiological pattern in patients with early RA. The initial DAS28 was slightly but significantly correlated with serum levels of beta2-microglobulin (*r *= 0.2, *P *<0.0001), IgG (*r *= 0.3, *P *<0.0001), IgA (*r *= 0.2, *P *<0.0001), IgM (*r *= 0.1, *P *= 0.002), and kappa and lambda FLCs (*r *= 0.3, *P *<0.0001 for both) but not BAFF, IgM-RF, IgA-RF, or anti-CCP. Initial HAQ was slightly but significantly correlated with all initial markers of B-cell activation except IgA-RF (data not shown). Among patients with early RA, levels of IgG, IgA, IgM, and kappa and lambda FLCs were significantly higher in early RA patients positive for IgM-RF or anti-CCP antibodies than in those negative for them (Table 3). IgA levels were higher in patients with IgA-RF than in those without IgA-RF (3.2 [2.5 to 3.9] versus 2.3 [1.7 to 2.9], *P *<0.0001). BAFF levels were similar regardless of the presence of autoantibodies (Table 3). Serum levels of BAFF, beta2-microglobulin, IgG, IgM, and lambda FLCs were not associated with the presence of radiological erosions at enrollment. Conversely, levels of total IgA and kappa FLCs were significantly higher in patients with erosion (3.0 [2.1 to 3.7] g/L and 15.3 [11.9 to 20.7] mg/L, respectively) than without erosion (2.6 [1.9 to 3.4] g/L and 13.9 [10.4 to 17.9] mg/L, *P *= 0.003 and *P *= 0.007). On univariate analysis, other parameters associated with radiological erosions at enrollment included IgM-RF, IgA-RF, anti-CCP, and ESR (Table 4). In the multivariate analysis, only anti-CCP (OR 2.3 [1.2 to 4.3], *P *= 0.008) and IgA-RF positivity (OR 1.7 [1.1 to 2.7], *P *= 0.01) were associated with early erosions (Table 4).

**Table 3:** Association between baseline B-cell activation biomarkers and immunological features of the 578 patients with early rheumatoid arthritis

	IgM RF +	IgM RF -	*P *value	Anti-CCP +	Anti-CCP -	*P *value
	n = 321	n = 257		n = 274	n = 304	
BAFF	0.7 (0.5–0.9)	0.7 (0.5–1.0)	0.18	0.7 (0.5–0.9)	0.7 (0.5–0.9)	0.18
Beta2–microglobulin	2.1 (1.8–2.4)	1.9 (1.6–2.3)	0.01	2.0 (1.7–2.4)	1.9 (1.6–2.4)	0.30
Total IgG	14.0 (12.0–16.9)	13.0 (11.1–15.1)	<10^-4^	14.4 (12.4–17.1)	12.9 (11.1–15.9)	<10^-4^
Total IgA	2.9 (2.1–3.7)	2.5 (1.9–3.3)	<10^-4^	3.1 (2.3–3.8)	2.4 (1.8–3.2)	<10^-4^
Total IgM	1.6 (1.2–2.2)	1.3 (1.1–1.8)	<10^-4^	1.6 (1.2–2.3)	1.4 (1.0–1.9)	<10^-4^
Kappa free light chain	15.8 (12.2–20.6)	12.7 (9.5–16.8)	<10^-4^	16.3 (12.7–20.6)	12.7 (9.7–17.1)	<10^-4^
Lambda free light chain	18.6 (14.4–25.0)	15.0 (11.8–19.6.)	<10^-4^	19.3(14.9–24.9)	15.1 (11.9–19.9)	<10^-4^

Results are expressed as median values (25th–75th values). B-cell activating factor of the tumor necrosis factor family (BAFF) values are expressed in nanograms per milliliter, total immunoglobulin values in grams per liter, and free light chain of immunoglobulins in milligrams per liter. Comparisons used Mann-Whitney *U *test. Anti-CCP, anti-cyclic citrullinated peptide; RF, rheumatoid factor.

**Table 4:** Baseline clinical and biological features in patients with early rheumatoid arthritis according to the presence of radiological erosions at enrollment

	Early erosions	Absence of early erosion	Univariate analysis: *P *value	Multivariate analysis: OR (95% CI), *P *value
	n = 138	n = 377		
Initial DAS28	5.4 (4.6–6.4)	5.2 (4.3–6.5)	0.1	
ESR, mm at 1st hour	29.5 (13.0–54.0)	20.5 (11.0–36.0)	0.002	1.1 (0.9–1.3), 0.4
CRP, mg/L	11.0 (4.0–33.1)	9.0 (0–22.0)	0.1	
IgM-RF-positive	63.7%	52.5%	0.03	0.7 (0.4–1.3), 0.2
IgA-RF-positive	64.5%	45.9%	0.0003	1.7 (1.1–2.7), 0.01
Anti-CCP-positive	61.6%	41.6%	<0.0001	2.3 (1.2–4.3), 0.008
BAFF, ng/mL	0.7 (0.5–0.9)	0.8 (0.5–1.0)	0.5	
Beta2–microglobulin, mg/L	2.1 (1.7–2.4)	1.9 (1.7–2.4)	0.08	
Total IgG, g/L	13.7 (12.0–15.6)	13.3 (11.5–15.9)	0.2	
Total IgA, g/L	3.0 (2.1–3.7)	2.6 (1.9–3.4)	0.003	1.1 (0.9–1.2), 0.4
Total IgM, g/L	1.4 (1.1–2.1)	1.5 (1.1–2.1)	0.9	
Kappa free light chain, mg/L	15.3 (11.9–20.7)	13.9 (10.4–17.9)	0.007	1.0 (0.9–1.1), 0.2
Lambda free light chain, mg/L	18.3 (13.5–23.4)	16.9 (12.8–21.9)	0.1	

Results are expressed as median values (25th–75th values) or as percentages. Comparison of proportions of presence of IgM-RF, IgA-RF, and anti-cyclic citrullinated peptide (anti-CCP) used chi-square test, and comparison of the other variables used Mann-Whitney *U *test. Baseline markers associated on univariate analysis with early erosions (radiological erosions at enrollment) were analyzed in multivariate analysis. BAFF, B-cell activating factor of the tumor necrosis factor family; CI, confidence interval; CRP, C-reactive protein; DAS28, disease activity score using 28 joint counts; ESR, erythrocyte sedimentation rate; OR, odds ratio; RF, rheumatoid factor.

## Discussion

These findings from ESPOIR, the prospective French multicenter cohort of early arthritis patients, demonstrate that serum markers of B-cell activation are elevated in patients with early RA compared with undifferentiated early arthritis. They also show that markers of B-cell activation are correlated with disease activity. Lastly, they indicate that serum BAFF might not drive the specific B-cell activation we observed in early RA. ESPOIR offered us the opportunity to investigate markers of B-cell activation in patients with early arthritis who had not yet been exposed to corticosteroids or DMARDs at enrollment. The disease-related increase of markers of B-cell activation might otherwise have been masked by these treatments, which can modulate these marker levels [16,17]. Another obvious advantage of performing such a study with this cohort was the centralization of biological, immunological, and radiological examinations and the prospective follow-up, which allows us to determine whether markers of B-cell activation can serve as predictive factors of long-term clinical activity.

The main limitations of this study were the short duration of follow-up (1 year) so far, since ACR criteria for RA might be fulfilled later, and the absence of validated criteria for early RA. Although the 1987 ACR criteria lack specificity for early RA diagnosis [18], they remain widely used in patients with early arthritis in the absence of any other international criteria for early RA. The percentage of patients who fulfilled these 1987 ACR criteria of RA is higher in the ESPOIR cohort (72% at inclusion and 79% at 1 year) than in other early arthritis cohorts [19,20]. This may be related to the inclusion criteria of ESPOIR, which were more stringent (at least two swollen joints and 6 weeks of duration) than those of some other early arthritis cohorts. It has been suggested that the specificity of ACR criteria in early RA may improve when the physician's level of confidence in the RA diagnosis is high [18]. At the ESPOIR 1-year follow-up visit, the rheumatologists completed a visual analog scale (VAS) assessing their confidence in the RA diagnosis for each patient. We were therefore able to conduct a separate analysis of the 367 of the 578 patients fulfilling 1987 ACR criteria for RA for whom this VAS was equal to or greater than 75 mm on a scale of 100 mm. Even when these more stringent criteria for RA were used, levels of IgG, IgA, and kappa FLCs remained higher in early RA than in undifferentiated early arthritis (data not shown). Lastly, since few serum markers of other immune cells are available in routine, only markers of B-cell activation were assessed in the present study, which did not address the pathogenic role of other immune cells.

The ESPOIR cohort confirms the pathogenic activation of autoreactive B cells in early RA, reflected by the association between anti-CCP and IgA-RF with early radiological erosions, also observed in previous early RA cohorts [21,22]. The originality of the present study is to also assess markers of activation of alloreactive B cells in early arthritis, such as total levels of immunoglobulins, FLCs of immunoglobulins, and beta2-microglobulin.

The first important finding is that serum level markers of alloreactive B-lymphocyte activation are higher in early RA than in UA. Some features of B-cell activation were nonetheless observed in patients with UA, including BAFF levels that were higher than in healthy donors, elevated IgG in 55% of the patients, and elevated levels of immunoglobulin FLCs in 22% (compared with 0% in healthy donors, according to previous studies [9,23]). During follow-up, it will be interesting to study whether patients with UA and high levels of markers of B-cell activation may more frequently develop RA or another autoimmune disease, such as systemic lupus erythematosus (SLE) or primary Sjögren syndrome, both of which feature elevated levels of markers of B-cell activation [9]. The markers we assessed reflect different stages of B-lymphocyte activation. All of them except IgM and BAFF were elevated in early RA. Beta2-microglobulin, present at the surface of all nucleated cells in association with major histocompatibility complex (MHC) class I molecules, is secreted mainly by B cells and plasma cells, while plasmablasts and plasma cells secrete immunoglobulin FLCs. Increased levels of the latter thus suggest that plasmablasts might also be activated in early RA. The normality of the kappa-to-lambda ratio in nearly all study patients confirmed that B-cell activation is polyclonal in RA. As opposed to IgM, IgG and IgA are secreted by mature plasma cells, which are a terminal stage of B-cell differentiation, after isotype class switching. A case-control study observed that, more than 10 years before RA development, future patients had elevated levels of IgG and IgA, but not of IgM, as in our patients with early RA [8].

Our second important finding was the absence of an association between the BAFF gene polymorphism and either RA or any of the baseline clinical, immunological, or radiological characteristics of RA. The lack of an association with RA is consistent with a previous Japanese genetic study [24]. We found no association between the BAFF polymorphism and serum BAFF levels, contrary to findings of our group and others in primary Sjögren syndrome and hematological disorders [12-14], in which the BAFF 871T>C allele was associated with higher serum levels of the cytokine. This discrepancy may be due to much higher serum BAFF levels in the diseases in which this association has been observed. The polymorphism may thus predispose patients to higher BAFF levels in diseases in which environmental stimulations trigger BAFF secretion.

Serum BAFF level was higher than in healthy blood donors, as previously reported for patients with established disease [25,26]. However, BAFF levels did not differ significantly between patients with early RA and those with undifferentiated early arthritis. Serum BAFF level was not associated with total Ig, RF, or anti-CCP levels, disease activity, or initial radiological erosions. These results are consistent with previous reports showing no difference in serum BAFF levels for patients with RA and other arthritides, including SLE, spondylarthropathy, crystal-induced arthritis, and osteoarthritis [26,27]. One study found an association between serum BAFF and early RA but in a much smaller population (n = 48) and with a different technique for BAFF assessment [28].

Thus, the specific increase of markers of B-cell activation in early RA might not be related to the increase of BAFF secretion. Membrane-bound BAFF or post-translational changes of BAFF (glycosylation, trimerization with a proliferation-inducing ligand [APRIL] or delta BAFF) might affect serum B-cell activation independently of the quantity of BAFF secreted and detected in the serum. The role of local BAFF production in arthritic joints cannot be ruled out either since BAFF levels are reported to be higher in synovial fluid than in serum [27]. As in serum, however, these levels in synovial fluid do not differ for patients with RA and with other inflammatory arthritides [27].

Our results thus suggest that serum BAFF does not drive B-cell activation in early RA, at least not directly or solely. The triggering forces of B-cell activation in early RA may depend on T lymphocytes. A specific pattern of T-lymphocyte activation was recently reported in RA, with Th2 (interleukin [IL]-4, IL-13) and IL-17 expression increasing temporarily in the synovial fluid of patients with very early RA [29]. Th2 profiles favor B-cell activation and antibody secretion. Among the factors unrelated to T cells, the role of innate immune cytokines, such as IL-6, should also be considered.

Interestingly, our results showed elevated serum IgA levels in early RA. This might be related to synovial, T cell-independent activation of B lymphocytes, which might be locally triggered by resident cells of autoimmunity, like synoviocytes, as described in mucosa [30]. In regard to the cytokine triggers of IgA increase, transforming growth factor-beta, which enhances IgA class switching [31], might be involved. T cell-independent class IgA class switching might also be favored by APRIL, a cytokine of the TNF family that shares two of the three receptors of BAFF: B-cell maturation antigen (BCMA) and TACI (transmembrane activator and CAML [calcium modulator and cyclophilin ligand] interactor) [10]. APRIL levels are elevated in synovial fluid from patients with established RA [27], and synovial expression of APRIL (but not of BAFF) is reported to be highest in patients with germinal center-like structures in the synovium, a form of lymphoid organization known to enhance B-cell activation [32]. Investigation of the role of APRIL in the B-cell activation of patients with early RA therefore appears useful.

Lastly, one of the important results of the study was the association observed between the increase in some markers of B-cell activation and disease activity (assessed by DAS28) and between the increase in some markers of B-cell activation (total IgA and kappa FLCs) and disease severity (early radiological erosion at inclusion). The relevance of markers of B-cell activation as prognostic markers will be further investigated throughout the cohort study, planned to last for 10 years to ensure assessment of patients' long-term clinical and radiological course. From a therapeutic perspective, these results suggest the potential value of B-cell depletion in early RA, which is currently under evaluation in controlled trials.

## Conclusions

The elevation of markers of B-cell activation in very early RA demonstrates that B-cell activation is an early pathogenic event in RA. B-cell activation is not directly driven by a systemic increase of BAFF in early RA. The increase of some markers of B-cell activation is associated with disease activity, autoantibody secretion, and early radiological erosion. This study thus sheds new light on the early pathogenic role of B lymphocytes in RA and suggests that targeting these cells might be a useful therapeutic strategy in early RA.

## Abbreviations

ACR: American College of Rheumatology; anti-CCP: anti-cyclic citrullinated peptide; APRIL: a proliferation-inducing ligand; BAFF: B-cell activating factor of the tumor necrosis factor family; CRP: C-reactive protein; DAS28: disease activity score using 28 joint counts; DMARD: disease-modifying antirheumatic drug; ELISA: enzyme-linked immunosorbent assay; ESR: erythrocyte sedimentation rate; FLC: free light chain; HAQ: Health Assessment Questionnaire; IL: interleukin; OR: odds ratio; RA: rheumatoid arthritis; RF: rheumatoid factor; SLE: systemic lupus erythematosus; TNF: tumor necrosis factor; UA: undifferentiated arthritis; VAS: visual analog scale.

## Competing interests

The authors declare that they have no competing interests.

## Authors' contributions

J-EG and XM analyzed the data and wrote the manuscript. CM-R analyzed BAFF gene polymorphism. BD performed statistical analyses. PG and BC contributed to the analysis and revised the manuscript. All authors read and approved the final manuscript.
